# Supplementary material for: Learning with reinforcement prediction errors in a model of the Drosophila mushroom body
Source: Nat Commun. 2021 May 7;12:2569. doi: 10.1038/s41467-021-22592-4 (PMC8105414; doi:10.1038/s41467-021-22592-4)
Supplement: Supplementary file 1 — Supplementary Information [file 41467_2021_22592_MOESM1_ESM.pdf]

Learning with reinforcement prediction errors in a model of the *Drosophila* mushroom body

Supplementary Information

James E. M. Bennett[1], Andrew Philippides[1], Thomas Nowotny[1]

[1]Department of Informatics, University of Sussex, UK

Corresponding author:

James E. M. Bennett

School of Engineering and Informatics

University of Sussex

Chichester 1, Room 002

Falmer

Brighton

BN1 9QJ

Supplementary Note

Mathematical solutions for experimental interventions that differentiate the VSλ and MV models

In Fig. 5d-e we highlighted an example experimental intervention that revealed substantial differences between the learned behaviour produced by the VSλ and the MV models. The intervention comprised appetitive conditioning with shibire block of M$+$ (approach MBON) in the training phase during both CS$+$ and CS$-$ exposure. In the following, we describe how the two models produce these different behaviours.

In this example, the VSλ model yielded a strong reduction in the preference for the CS$+$ as compared with controls, whereas there was a small positive increase in the CS$+$ preference in the MV model. There are two factors at play here, which we outline and then backup by analysing the equations for the synaptic weight dynamics. First, inhibitory block of M$_+$ during training in the MV model amplifies the difference between reinforcement predictions (RPs) for the CS$+$ and CS$-$, $\hat{m}_{\mathrm{CS}+} - \hat{m}_{\mathrm{CS}-}$, whereas in the VS$\lambda$ model, $\hat{m}_{\mathrm{CS}+} - \hat{m}_{\mathrm{CS}-}$ is the same as in the control. Second, $\hat{m}_{\mathrm{CS}+}$ decays to zero more rapidly in the VSλ model than in the MV model during the test phase. Combining this second point with the fact that $\hat{m}_{\mathrm{CS}+} - \hat{m}_{\mathrm{CS}-}$ is smaller in the VSλ model implies that, over the two testing trials in our simulations, $\hat{m}_{\mathrm{CS}+} - \hat{m}_{\mathrm{CS}-}$ diminishes to zero very quickly in the VSλ model, resulting in a reduced preference for the CS$+$. Because $\hat{m}_{\mathrm{CS}+} - \hat{m}_{\mathrm{CS}-}$ is amplified in the MV model, its preference for the CS$+$ remains strong, and even appears to increase by a small amount, resulting in only a small, positive Δ_f.

To explain the effect of the inhibitory block on the magnitude of $\hat{m}_{\mathrm{CS}+} - \hat{m}_{\mathrm{CS}-}$,

we analyse the synaptic weight dynamics, $\dot{\mathrm{w}}_{\pm}$, given the reinforcement provided ($r_{+} = 1$, $r_{-} = 0$) and the inhibition of M_{+}. In the VSλ model, the weight dynamics are given by:

$$\begin{aligned}
\dot{\mathbf{w}}_{\pm}^{\mathrm{VS}\lambda} &= \eta\left(\lambda - d_{\mp}\right) \\
\dot{\mathbf{w}}_{+}^{\mathrm{VS}\lambda}\Big|_{\mathrm{CS}+} &= \eta\left(\lambda - 0.1m_{+} - \mathbf{w}_{\mathrm{K}}\mathbf{k}\right) && (27) \\
\dot{\mathbf{w}}_{-}^{\mathrm{VS}\lambda}\Big|_{\mathrm{CS}+} &= \eta\left(\lambda - r_{+} - m_{-} - \mathbf{w}_{\mathrm{K}}\mathbf{k}\right) && (28) \\
\dot{\mathbf{w}}_{+}^{\mathrm{VS}\lambda}\Big|_{\mathrm{CS}-} &= \eta\left(\lambda - 0.1m_{+} - \mathbf{w}_{\mathrm{K}}\mathbf{k}\right) && (29) \\
\dot{\mathbf{w}}_{-}^{\mathrm{VS}\lambda}\Big|_{\mathrm{CS}-} &= \eta\left(\lambda - m_{-} - \mathbf{w}_{\mathrm{K}}\mathbf{k}\right), && (30)
\end{aligned}$$

and for the MV model, we have:

$$\begin{aligned}
\dot{\mathbf{w}}_{\pm}^{\mathrm{MV}} &= \frac{\eta}{2}\left(\mathbf{w}_{\mathrm{K}}\mathbf{k} - d_{\mp}\right) \\
\dot{\mathbf{w}}_{+}^{\mathrm{MV}}\Big|_{\mathrm{CS}+} &= \frac{\eta}{2}\left(r_{+} - \left(0.1m_{+} - m_{-}\right)\right) && (31) \\
\dot{\mathbf{w}}_{-}^{\mathrm{MV}}\Big|_{\mathrm{CS}+} &= \frac{\eta}{2}\left(-r_{+} - \left(m_{-} - 0.1m_{+}\right)\right) && (32) \\
\dot{\mathbf{w}}_{+}^{\mathrm{MV}}\Big|_{\mathrm{CS}-} &= \frac{\eta}{2}\left(m_{-} - 0.1m_{+}\right) && (33) \\
\dot{\mathbf{w}}_{-}^{\mathrm{MV}}\Big|_{\mathrm{CS}-} &= \frac{\eta}{2}\left(0.1m_{+} - m_{-}\right), && (34)
\end{aligned}$$

where the factor of $1/2$ compensates for the fact that each lobe in the MV model learns in proportion to the full RPE signal, whereas each lobe in the MVλ model learns in proportion to half of the RPE signal. Given sufficient time for the synaptic weights to stabilise during learning, such that $\dot{\mathrm{w}}_{\pm} = 0$, we can write expressions for the steady state values of m_{+} and m_{-}, from which we can calculate the relative RPs for the CS$+$ and CS$-$, and thereby determine the strength of appetitive or aversive behaviour in the two models. In the VSλ model, we obtain for the MBON

firing rates:

$$\begin{aligned} m_{+}^{\mathrm{VS}\lambda}\big|_{\mathrm{CS}+} &= 10c \\ m_{-}^{\mathrm{VS}\lambda}\big|_{\mathrm{CS}+} &= c - r_{+} \\ m_{+}^{\mathrm{VS}\lambda}\big|_{\mathrm{CS}-} &= 10c \\ m_{-}^{\mathrm{VS}\lambda}\big|_{\mathrm{CS}-} &= c, \end{aligned}$$

where $c = \lambda - \mathbf{w}_{\mathrm{K}}\mathbf{k}$ is a constant. As such, the difference between CS$+$ and CS$-$ RPs is:

$$\begin{aligned} \hat{m}_{\mathrm{CS}+}^{\mathrm{VS}\lambda} - \hat{m}_{\mathrm{CS}-}^{\mathrm{VS}\lambda} &= 10c - (c - r_{+}) - (10c - c) \\ &= r_{+}. \end{aligned} \tag{35}$$

That is, the net preference for the CS$+$ over the CS$-$ does not change, as the RP for both is increased by the same amount due to the inhibition of M_{+}.

In the MV model, however, both DANs process the r_{+} reinforcement signal. As such, inhibition of M_{+} influences the firing rates of both M_{+} and M_{-} as follows:

$$\begin{aligned} m_{+}^{\mathrm{MV}}\big|_{\mathrm{CS}+} &= 10\,(r_{+} + m_{-}) \\ m_{-}^{\mathrm{MV}}\big|_{\mathrm{CS}+} &= 0.1m_{+} - r_{+} \\ m_{+}^{\mathrm{MV}}\big|_{\mathrm{CS}-} &= 10m_{-} \\ m_{-}^{\mathrm{MV}}\big|_{\mathrm{CS}-} &= m_{-}. \end{aligned}$$

and consequently, the difference between CS_{+} and CS_{-} RPs in the MV model is also affected:

$$\begin{aligned} \hat{m}_{\mathrm{CS}+}^{\mathrm{MV}} - \hat{m}_{\mathrm{CS}-}^{\mathrm{MV}} &= 10\,(r_{+} + m_{-}) - 0.1 \times 10\,(r_{+} + m_{-}) + r_{+} - (10m_{-} - m_{-}) \\ &= 10r_{+}. \end{aligned} \tag{36}$$

Thus, approach behaviour towards the CS$+$ is ten times stronger in the MV model than in the VSλ model. This difference in CS$+$ preference cannot, by itself, explain the behaviours exhibited by the two models. This is because, in our simulations, behaviour is measured in the test phase across two trials, such that learning after the first trial may affect the behaviour in the second trial. Thus, the rate at which the appetitive memory is forgotten in the test phase must also be considered.

The rate of memory decay during the test phase is given by the difference in the synaptic weight dynamics of $\mathbf{w}_+$ and $\mathbf{w}_-$, taking into account that M$+$ is no longer receiving inhibitory block, and that $r_+ = 0$. Given that, in both the VSλ and MV models, the CS$+$ is preferred over the CS$-$, we assume that the CS$+$ is chosen in the first trial of the test phase in both models (which is almost always true in the simulations). We therefore only need to calculate the decay of the CS$+$ memory between trials 1 and 2. For the VSλ model, this is:

$$\begin{aligned}
\dot{\hat{\mathbf{w}}}^{\mathrm{VS}\lambda}_{\mathrm{CS}+} &= \dot{\mathbf{w}}^{\mathrm{VS}\lambda}_{+} - \dot{\mathbf{w}}^{\mathrm{VS}\lambda}_{-} \\
&= \eta\mathbf{k}\left[(\lambda - m_+ - \mathbf{w}_{\mathrm{K}}) - (\lambda - m_- - \mathbf{w}_{\mathrm{K}})\right] \\
&= \eta\mathbf{k}\,(m_- - m_+) \\
&= \eta\mathbf{k}\,(c - r_+ - 10c) \\
&= -19\,\eta\mathbf{k},
\end{aligned}$$

where we have substituted in the stabilised MBON firing rates, as calculated earlier, and used the following parameters from the simulations: $c = 2$ ($\lambda = 12$, $\mathbf{w}_{\mathrm{K}}\mathbf{k} = 10$) and $r_+ = 1$ (from the training phase).

The decay rate of the CS$+$ memory in the MV model is given by

$$
\begin{aligned}
\dot{\hat{\mathbf{w}}}_{\mathrm{CS}+}^{\mathrm{MV}} &= \dot{\mathbf{w}}_{+}^{\mathrm{MV}} - \dot{\mathbf{w}}_{-}^{\mathrm{MV}} \\
&= \frac{\eta}{2}\mathbf{k}\left[(m_{-} - m_{+}) - (m_{+} - m_{-})\right] \\
&= \eta\mathbf{k}\left(m_{-} - m_{+}\right) \\
&= \eta\mathbf{k}\left(m_{-} - 10\left(r_{+} + m_{-}\right)\right) \\
&= -10\,\eta\mathbf{k},
\end{aligned}
$$

where we have assumed that, before training, $\mathbf{w}_{\pm}^{\mathrm{MV}} = \epsilon$, with ϵ a small number $\ll 1$, such that $\mathbf{w}_{-}^{\mathrm{MV}}$ were sufficiently low to have reached zero during training (as was the case in our simulations).

We have found that the CS$+$ memory in the VSλ model decays almost two times faster than in the MV model. This result, in combination with the fact that the difference between CS$+$ and CS$-$ RPs in the VSλ model is smaller than in the MV model, tells us that choice behaviour in the VSλ model becomes more random much sooner than in the MV model, resulting in the strong relative decrease in CS$+$ choices as shown in Fig. 5d-e.

Supplementary Discussion

Relationship between RPE coding in our model and experimental observations of DAN physiology

In vertebrates, DANs in the midbrain exhibit signals that are indicative of reinforcement prediction error (RPE) coding [1]. These RPE signals include two salient characteristics: i) a reduction in the US response amplitude after conditioning, and ii) an increase in the CS$+$ response amplitude after conditioning.

Using an aversive conditioning experiment in which odours were paired with electric shock, Riemensperger et al. [2] provided the first clear evidence that DANs in *Drosophila* carried reinforcing information. Riemensperger et al. also found that CS$+$ responses in DANs became more sustained after conditioning, suggesting they carried predictive information about the US. However, they found no significant change in DAN response amplitudes to either the CS$+$ or to the US after conditioning, which are hallmark characteristics of RPE coding. While their work has not yielded evidence for RPE coding, it may be premature to exclude the possibility of RPE coding in *Drosophila* based on this work alone, for both theoretical and experimental reasons, which we discuss below.

Theoretical considerations

The characteristics of RPE coding in mammals are typically linked with the theory of temporal difference (TD) learning [3–5], in which TD errors bear similarities to RPEs. Indeed, the Rescorla-Wagner rule [6] used in our work is a simplified case of the TD learning rule. Applying the TD learning rule to our MB model, the TD error at time t, δ_t, can be expressed as:

$$\delta_{t-1} = \hat{r}_t + \gamma_{\mathrm{TD}} \hat{m}_t - \hat{m}_{t-1}, \tag{37}$$

where $\hat{r}$ and $\hat{m}_t$ are respectively the reinforcement and reinforcement prediction (RP) at time t, and δ_{t-1} is the TD error used to update synaptic weights from KCs that were active in the previous time step. Thus, δ_{t-1} updates RPs relating to cues that precede, or predict, any TD errors. In TD learning, future reinforcements are discounted by an amount set by γ_{TD}. When $\gamma_{\mathrm{TD}} = 0$, the TD rule is equivalent to the RW rule, and only immediate reinforcements are learned. When $\gamma_{\mathrm{TD}} = 1$, the

TD rule learns to predict reinforcements that occur at any time in the future. As such, TD learning can form associations between a CS$+$ and US, even if they do not occur together. As $\gamma_{\rm TD}$ is decreased from 1 to 0, the temporal horizon within which a US can be associated with a CS$+$ diminishes. Consequently, smaller values of $\gamma_{\rm TD}$, and longer intervals between the CS$+$ and US, both diminish the learned increase in DAN responses to the CS$+$. If *Drosophila* heavily discount future reinforcements – i.e. if $\gamma_{\rm TD}$ were small – one would not expect to see a significant increase in the DAN response to the CS$+$ after conditioning. This feature of TD learning provides a possible explanation for why Riemensperger et al. did not observe a statistically insignificant increase in DAN responses to the CS$+$. Also note that, in a more recent study, Dylla et al. [7] did observe learning induced increases in DAN responses to a CS$+$. We discuss the results from Dylla et al. in more detail in the Discussion of the main text.

Experimental considerations

Several experimental studies [2; 7; 8] that looked for changes in DAN responses after conditioning used a TH-GAL4 line to label DANs in the mushroom body. DANs labelled by TH-GAL4 are now known to encode aversive as well as appetitive stimuli [9; 10], making it difficult to disentangle the expected response changes in both populations of DANs. Moreover, there are additional sources of heterogeneity across the labelled DANs. For example, optogenetic stimulation of V2 MBONs inhibits or excites different DANs in the PPL1 cluster (which the TH-GAL4 line labels; see Fig. 3 in [11]), and odour responses in PPL1 DANs vary considerably [8]. It is possible that not all DANs exhibit RPE signals, and such heterogeneity may well obscure their visibility when imaging from the whole population labelled by TH-GAL4. Transient RPE signals may be further obscured by the slow dynamics that are characteristic of fluorescent calcium indicators, and that are induced by the durations (seconds to minutes) of CS and US presenta-

tions. The discovery and use of genetic lines that more precisely target specific DANs in the MB, coupled with the use of calcium indicators or electrophysiological recordings that have faster dynamics, would help to better elucidate the response properties of DANs and their contribution, if any, to RPE coding in *Drosophila*.

DAN responses to the CS$+$ in the VSλ and MV models

Consider the case in which our models undergo a phase of aversive conditioning (with $r_- = 1$, $r_+ = 0$), followed by a test phase in which the CS$+$ is presented without a US ($r_- = 0$). The plasticity rules we present in the main text specify that, during aversive conditioning, $\mathbf{w}_+$ will undergo synaptic depression (and $\mathbf{w}_-$ will undergo potentiation in the MV model). As the RPE is minimised, the D$_+$ and D$_-$ responses will tend towards a baseline firing rate that is determined by $\mathbf{w}_\mathrm{K}\mathbf{k}$, the direct input to DANs from KCs. Thus, after the initial excitatory response to the punishment, the D$_-$ response will decrease over the following trials (in the MV model, the D$_+$ firing rate will increase after its initial inhibited response). As such, when the punishment is later removed, and the CS$+$ is presented alone, D$_-$ will exhibit a reduced response compared to baseline (that is, an excitatory response that is weaker than $\mathbf{w}_\mathrm{K}\mathbf{k}$). This appears to contradict observations by Dylla et al. [7] when using the same experimental protocol, namely that DAN responses to the CS$+$ increased after conditioning. However, one of the simplifications in our model makes it difficult to draw comparisons with Dylla et al. Because we did not simulate time within a trial, the CS$+$ and US always occurred together. Thus, when the reinforcement goes from $r_- = 1$ to $r_- = 0$, one may equally interpret the decreased D$_-$ response in our model in one of two ways: i) as a response to the presentation of the CS$+$ alone, or ii) a response to the combined CS$+$ and omitted punishment. The latter would bear resemblance to the decreased firing rate in reward encoding DANs after reward was omitted following appetitive conditioning in mammals [5]. It is worth noting that temporal difference (TD) learning

[3], described above, of which the Rescorla-Wager rule used in our models is a simplified case, does provide a mechanism for increased CS$+$ responses and decreased US responses in DANs [4]. Thus, the difference between our results and those of Dylla et al. need not conflict with the RPE hypothesis *per se*, but may come down to limitations in our implementation of the model. How TD learning may be implemented by the MB is not yet clear, and goes beyond the scope of our current work. We speculate that it may be mediated via multiple feedback pathways between MBONs and DANs [12].

Plasticity rules for the MV model

In the main text, we describe two plasticity rules for the MV model that help to minimise the full cost function, C^{RPE}. Here, we outline some of the issues with the first plasticity rule that was described by Eq. 7:

$$\mathcal{P}_{\pm}^{\mathrm{MV}} = \eta \mathbf{k} \left(\mathbf{w}_{\mathrm{K}}^{\mathrm{T}} \mathbf{k} - d_{\mp} \right).$$

Eq. 7 specifies that each synapse requires information from only a single DAN, whereby the synapse and the DAN have opposite valences (e.g. $\mathbf{w}_{+}$ and D$_{-}$). However, it does not seem feasible that KC$\rightarrow$MBON synapses would have access to information about the excitatory current, $\mathbf{w}_{\mathrm{K}}^{\mathrm{T}}\mathbf{k}$, elicited by KCs in DANs. A simple heuristic solution would be to replace $\mathbf{w}_{\mathrm{K}}^{\mathrm{T}}\mathbf{k}$ with a free parameter, λ, as we had done for the VSλ model, and assume this to be a property of the synapse that can be tuned. However, this introduces a new problem of instability in the synaptic weights. When RPs accurately match the reinforcement, $\hat{r} = \hat{m}$, and thus plasticity should cease, the term in parentheses in the plasticity rule equals $\lambda - \mathbf{w}_{\mathrm{K}}^{\mathrm{T}}\mathbf{k}$. Thus, if $\lambda > \mathbf{w}_{\mathrm{K}}^{\mathrm{T}}\mathbf{k}$, both $\mathbf{w}_{+}$ and $\mathbf{w}_{-}$ increase linearly with time, or decrease to zero if $\lambda < \mathbf{w}_{\mathrm{K}}^{\mathrm{T}}\mathbf{k}$.

The final issue that arises with Eq. 7, and described in the main text, is that it does not reproduce experimental results in which artificial stimulation of either D_+ (or D_-) can act as a proxy for reward (or punishment) to induce a memory. The explanation for this is provided in Supplementary Fig. 4.

169 # Supplementary Figures

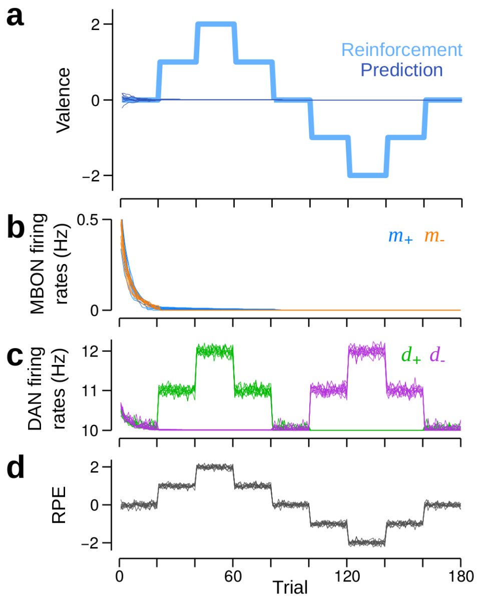

Supplementary Figure 1: Learning RPs in the valence-specific model with the derived plasticity rule given by $\mathcal{P}_{\pm}^{\mathrm{VS}}$ in Eq. 5 in the main text. **a** reinforcement schedule (excluding the Gaussian white noise; light blue) and RPs (dark blue) from 10 separate runs of the model. RPs in this model necessarily go to zero, because MBON firing rates also necessarily go to zero, as in **b**. **b** Firing rates of the M_+ (blue) and the M_- MBONs (orange). **c** Firing rates for the D_+ (green) and the D_- (purple) DANs in response to the reinforcement schedule in **a**. **d** Because RPs are always zero, RPEs, as given by the difference in firing rates of D_+ and D_-, are equal to the reinforcements themselves. Abbrev.: RP (reinforcement prediction); MBON (mushroom body output neuron); DAN (dopamine neuron); RPE (reinforcement prediction error); m_+, m_- (approach/avoidance MBON firing rate); d_+, d_- (appetitive/aversive DAN firing rate).

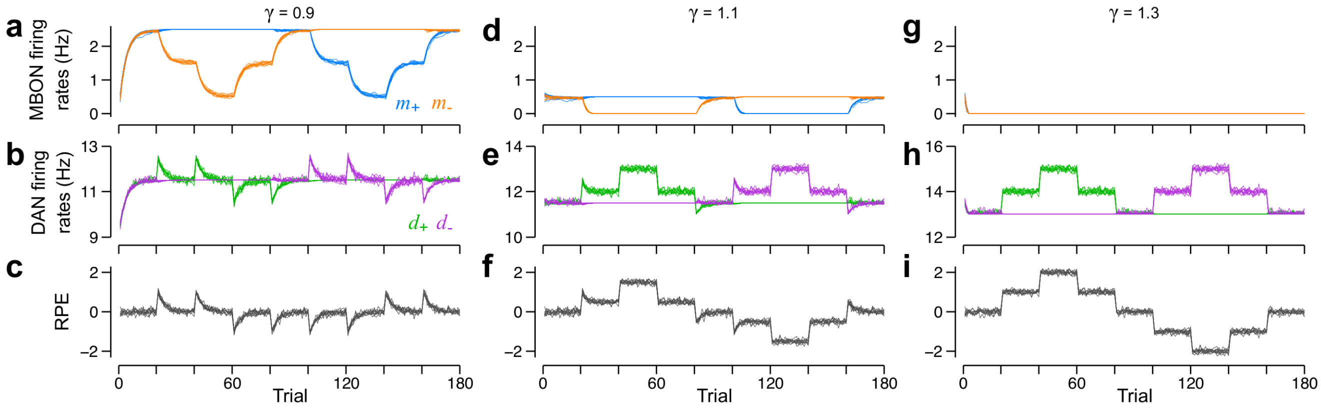

Supplementary Figure 2: The range of RPs that can be learned in the VSλ model increases as KC→DAN synaptic weights are weakened. Each column corresponds to the behaviour of the model with different KC→DAN synaptic weights, as specified by the γ value above each column. **a-c** $\gamma = 0.9$. **d-f** $\gamma = 1.1$. **g-i** $\gamma = 1.3$. The larger the value of γ, the greater the restriction to the range of MBON firing rates (**a**, **d**, **g**). As such, learning induced changes in MBON firing rates do not fully offset changes in DAN firing rates that follow the reinforcement signal (**b**, **e**, **h**). Consequently, lose their ability to signal RPEs (**c**, **f**, **i**). Abbrev.: RP (reinforcement prediction); MBON (mushroom body output neuron); DAN (dopamine neuron); RPE (reinforcement prediction error); m_+, m_- (approach/avoidance MBON firing rate); d_+, d_- (appetitive/aversive DAN firing rate).

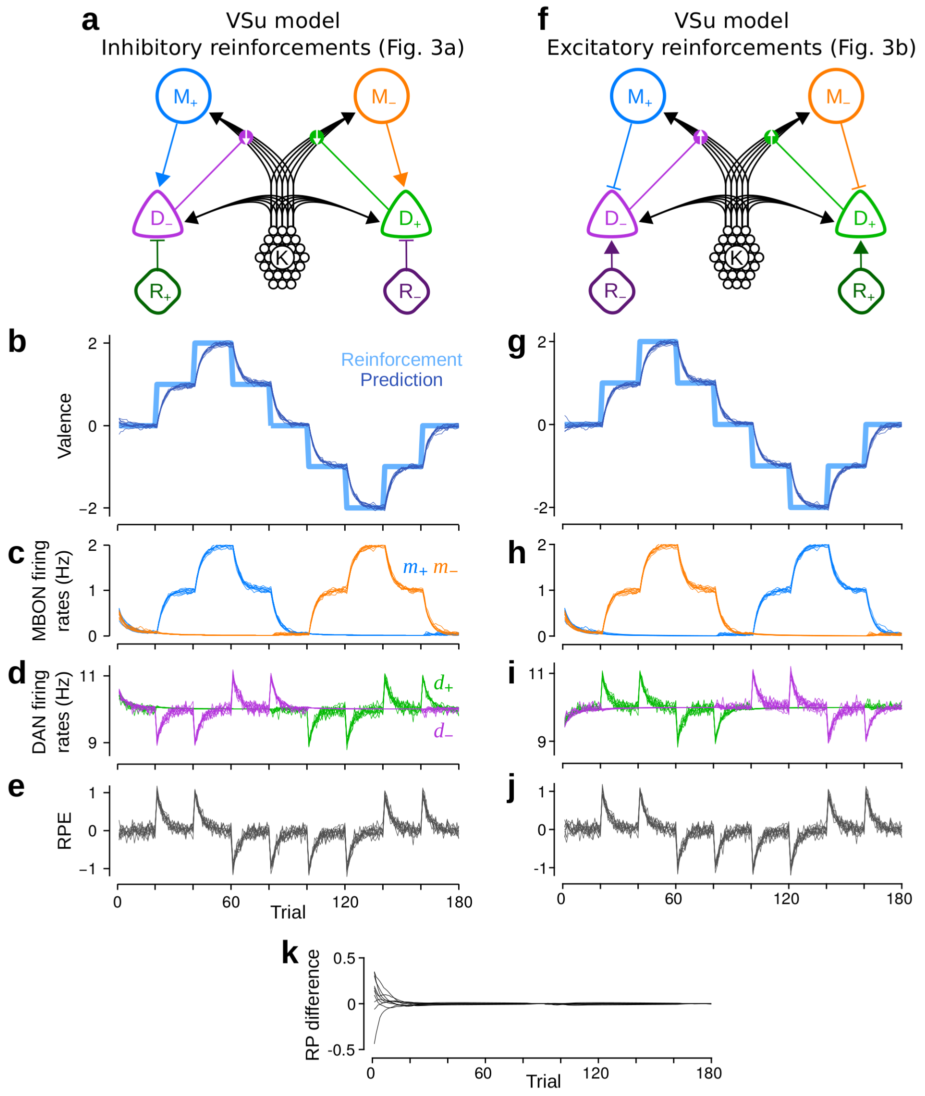

Supplementary Figure 3: Learning RPs in the dual valence-specific models with unbounded learning (VSu models, shown in Fig. 3a-b in the main text). The mixed-valence (MV) model is built by combining these dual models. **a** Circuit diagram for the VSu model with inhibitory reinforcements (as in Fig. 3a in the main text). This model uses the derived plasticity rule, $\mathcal{P}_{\pm}^{\mathrm{VS}}$ in Eq. 5). **b**-e Behaviour from the VSu model with inhibitory reinforcements. **b** Reinforcement schedule (excluding the Gaussian white noise; light blue) and RPs (dark blue) from 10 separate runs of the model. **c** Firing rates of the M_{+} (blue) and the M_{-} MBONs (orange). **d** Firing rates for the D_{+} (green) and the D_{-} (purple) DANs in response to the reinforcement schedule in **b**. **e** RPEs, as given by the difference in firing rates of D_{+} and D_{-}. **f** Circuit diagram for the VSu model with excitatory reinforcements (as in Fig. 3b in the main text). This model requires the plasticity rule, $\mathcal{P}_{\pm}^{\mathrm{VS}}$, to induce potentiation with elevated DAN firing rates. As such, the factor $(\mathbf{w}_{\mathrm{K}}^{\mathrm{T}}\boldsymbol{k} - d_{\mp})$ in $\mathcal{P}_{\pm}^{\mathrm{VS}}$ is replaced with $(d_{\mp} - \mathbf{w}_{\mathrm{K}}^{\mathrm{T}}\boldsymbol{k})$. **g**-j Same as for panels **b**-**e**, showing behaviour from the VSu model with excitatory reinforcements. Because the MBONs are inhibitory, M_{+} (M_{-}) inhibits approach (avoidance). RPs are therefore calculated using $\hat{m} = m_{-} - m_{+}$ for this model. **k** The difference between RPs learned in both VSu models, showing that each model learns nearly identical RPs. Early differences are due to the weights, $\mathbf{w}_{\pm}$, being initialised with random values. Abbrev.: RP (reinforcement prediction); MBON (mushroom body output neuron); DAN (dopamine neuron); RPE (reinforcement prediction error); m_{+}, m_{-} (approach/avoidance MBON firing rate); d_{+}, d_{-} (appetitive/aversive DAN firing rate).

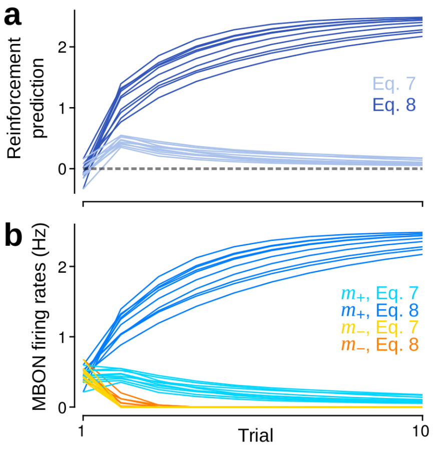

Supplementary Figure 4: Artificially stimulating DANs in the MV model induces a strong association if synaptic weight updates are governed by Eq. 8 (proportional to $d_{\pm} - d_{\mp}$). If weight updates are instead governed by Eq. 7 (proportional to $\mathbf{w}_{\mathrm{K}}\mathbf{k} - d_{\mp}$), only a weak association is induced, which disappears with repeated CS+-stimulation pairings. **a**) Reinforcement predictions after pairing the CS+ with D_{+} stimulation by adding an additional input to the D_{+} firing rate equation:

$$d_{+} = r_{+} - r_{-} - (w_{\mathrm{M}} m_{+} - w_{\mathrm{M}} m_{-}) + 5.$$

b) Both MV model plasticity rules depress the M_{-} firing rate after D_{+} stimulation. The brief, weak association that is induced with Eq. 7 disappears because feedback inhibition from M_{-} to D_{-} is reduced. Consequently, the D_{-} firing rate increases and the M_{+} firing rate is depressed as well. In contrast, when D_{+} modulates plasticity at both $\mathbf{w}_{+}$ and $\mathbf{w}_{-}$, as required by Eq. 8, D_{+} stimulation depresses the firing rate of M_{-} and potentiates the firing rate of M_{+}.

Abbrev.: MV (mixed valence); MBON (mushroom body output neuron); DAN (dopamine neuron); m_{+}, m_{-} (approach/avoidance MBON firing rate); d_{+}, d_{-} (appetitive/aversive DAN firing rate); $\mathbf{k}$ (Kenyon cell firing rate vector); r_{+}, r_{-} (reward/punishment amplitudes); w_{X} (synaptic weight from neuron type X).

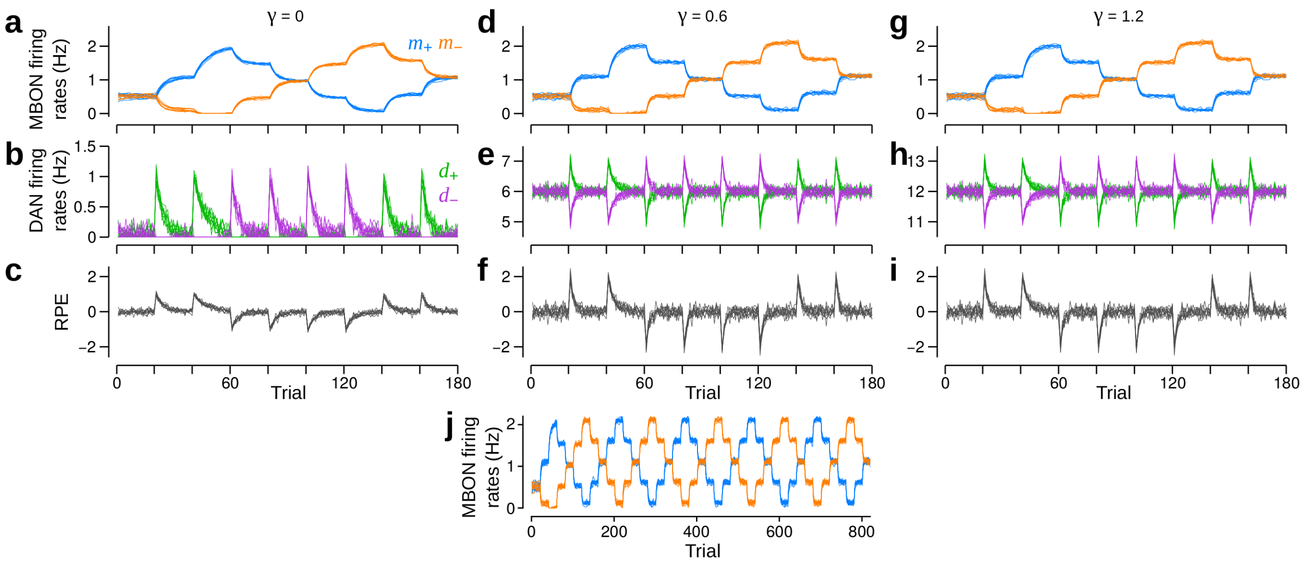

Supplementary Figure 5: Learning reinforcement predictions in the mixed-valence model. Each column corresponds to the behaviour of the model with different KC→DAN synaptic weights, as specified by the γ value above each column. **a**-**c** When γ is chosen such that $\mathbf{w}_{\mathrm{K}}^{\mathrm{T}}\mathbf{k}$ is less than the RPE magnitude, the dynamic range of DANs is restricted (**b**). As such, the RPE reported by the difference in firing rates of D_+ and D_- is reduced (**c**), resulting in slower learning (**a**), and RPEs that decay more slowly. **d**-**i** Above a critical value, changing the KC→DAN synaptic weights has no effect on model behaviour. **j** Because KC→MBON weights, $\mathbf{w}_\pm$, are initialised with small values, they must initially become larger on average in order to learn accurate RPs. Consequently, the mean MBON firing rates also increase initially (the trend of increasing firing rates up to trial 200), but then stabilise (after trial 200). Abbrev.: RP (reinforcement prediction); MBON (mushroom body output neuron); DAN (dopamine neuron); RPE (reinforcement prediction error); m_+, m_- (approach/avoidance MBON firing rate); d_+, d_- (appetitive/aversive DAN firing rate).

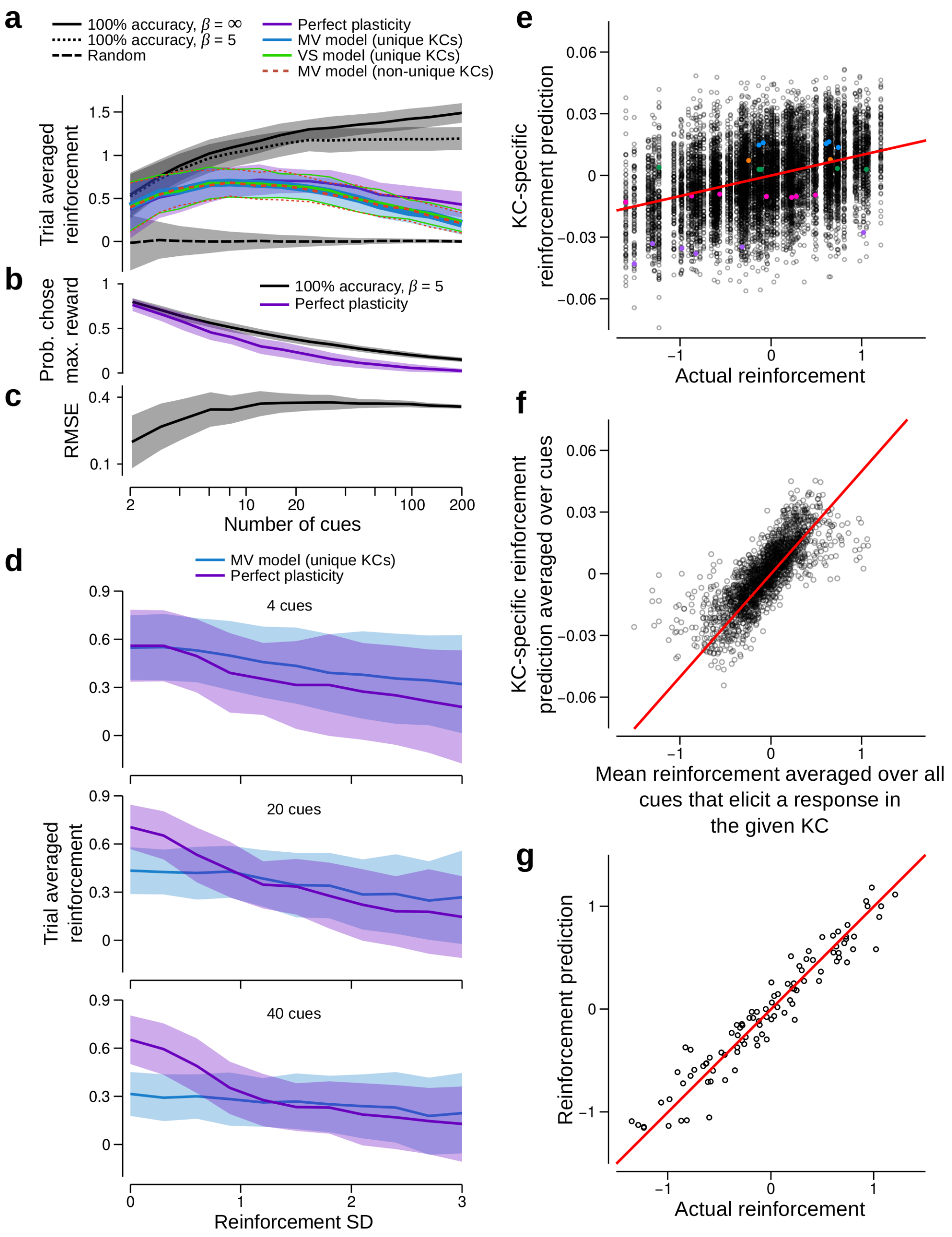

Supplementary Figure 6: Model performance, as compared with different idealised agents, for a multiple-alternative forced choice task involving 2 or more cues.

a The performance of the mixed valence (MV) model (blue line) and the valence specific (VSλ) model (green line), as measured by the trial averaged reinforcement (TAR) obtained, and as a function of the number of cues. As a comparison, we also provide the TAR for a perfect agent that always chooses the most positively reinforcing cue (black, solid curve) – this emulates an agent that predicts reinforcements for all cues with 100% accuracy, and is fully deterministic ($\beta = \infty$) – and the mean TAR for an agent that randomly chooses cues with equal probability for each cue (black, long-dashed curve). The TAR for the VSλ and MV models peaks when there are six cues to choose. This is likely due to the trivial fact that there are more positive reinforcements from which to choose when there are more cues, as demonstrated by the monotonic increase in TAR for the perfect agent.

A fairer comparison against the model (black, dotted curve) is an agent that predicts reinforcements for all cues with 100% accuracy, yet makes decisions probabilistically using the same softmax function used by the model ($\beta = 5$). This results in only a small reduction in the TAR compared to the perfect agent.

An even more realistic comparison (purple curve) is an agent that can only update reinforcement predictions (RPs) for the chosen cue, but otherwise exhibits perfect synaptic plasticity, i.e. the RP is set to be equal to the reinforcement just obtained for a given cue. This substantially diminishes performance, which becomes comparable to the model when the number of cues is fewer than twenty. We conclude that the reduced performance of the model, as compared with the 100% accurate agent, is due to the unavoidable fact that the model's ability to maintain accurate predictions for all cues decreases with the number of cues (panel **c**). This is exacerbated by the slowness of synaptic plasticity, as set by the learning rate. *Continued on next page.*

Supplementary Figure 6: *Continued from previous page.*
Finally, we tested a version of the model in which KC responses to different cues may overlap (red dashed line). This model comprised 2000 KCs, similar to estimates for a single hemisphere in *Drosophila* [13], of which ∼5% (totalling 100 KCs) were randomly selected to respond to each cue [14]. This means that, when there are 100 cues from which to choose, for example, each KC responds to 5 cues on average. Strikingly, this had negligible effect on the mean TAR obtained by the model. This may be surprising, as synapses from each KC end up learning the mean reinforcement across those 5 cues (panel **e**). However, because the reinforcement predicted for any one cue is encoded in the synapses from 100 KCs, the weighted input from all KCs to M_+ and M_- yields accurate RPs. We analyse this feature in greater detail in panels **e**-**g**. All lines: mean TAR across 100 simulation runs; shaded regions: standard deviation in the TAR.

b Probabilities of choosing the most rewarding cue for two of the ideal models discussed in **a**. Black line: the probability as computed using the softmax function with $\beta = 5$, for an agent that has full knowledge of all available reinforcements for the current trial (100% accurate RPs). Purple line: fraction of trials in which the perfect plasticity model actually chose the most rewarding cue. Lines: mean across 100 simulation runs; shaded regions: standard deviation

c Root mean squared error between RPs and actual reinforcements, where the mean is taken across all cues and all trials. Line: mean across 100 simulation runs; shaded region: standard deviation.

d A slow learning rate is advantageous when the reinforcements have a stochastic element. We added zero-mean Gaussian white noise, with SD σ_ξ, to the same, low-pass filtered reinforcement schedules. The performance of both the MV model and the agent with perfect plasticity decreased with increasing noise. However, the addition of stochastic noise had a much greater impact on the perfect plasticity agent. Any advantage it had over the MV model was lost for $\sigma_\xi \gtrsim 1.0$. Moreover, the performance of the agent was marginally, though consistently lower than the model in this regime.

e-g When cues elicit overlapping responses in KCs, accurate RPs may be obtained from the ensemble of KC inputs to the MBONs, despite the fact that each KC responds to multiple cues. Here, the MV model has 2000 KCs and is trained on 200 cues. RPs are updated on each trial for all cues, rather than for the chosen cue. All reinforcements and RPs are taken from the final trial in the simulation, after learning has converged.

e Each data point corresponds to the reinforcement for a single cue, and a single KC's contribution (the difference between excitatory currents it elicits in M_+ and M_-) to the RP for that cue. The contribution from individual KCs to the overall RP is very noisy (variability in the vertical axis). This is because the weight from each KC can only learn the mean reinforcement over the cues to which that KC responds. RP contributions from five example KCs are shown in the coloured data points (each colour corresponds to a single KC, and each KC responds to multiple cues). The RP contribution from each KC is approximately the same for all cues to which it responds. Note that, because each cue elicits a response in 100 KCs on average, the RP contribution from each KC is approximately 1% of the total RP. The red line plots $y = x/100$ to show this correspondence between reinforcements and KC-specific RPs.

f A single KC's contribution to the RP, averaged across all cues to which it responds, corresponds well with approximately 1% of the actual reinforcement averaged across those cues. KCs in these simulations respond to 5 cues, on average. The red line shows the expected correspondence, and plots $y = 5x/100$.

g Although the contribution from each KC toward the total RP is noisy, the total RP, which is the summed contributions over all responding KCs, is relatively precise. Red line: line of equality.

Abbrev.: MV (mixed valence); VS (valence specific); KC (Kenyon cell).

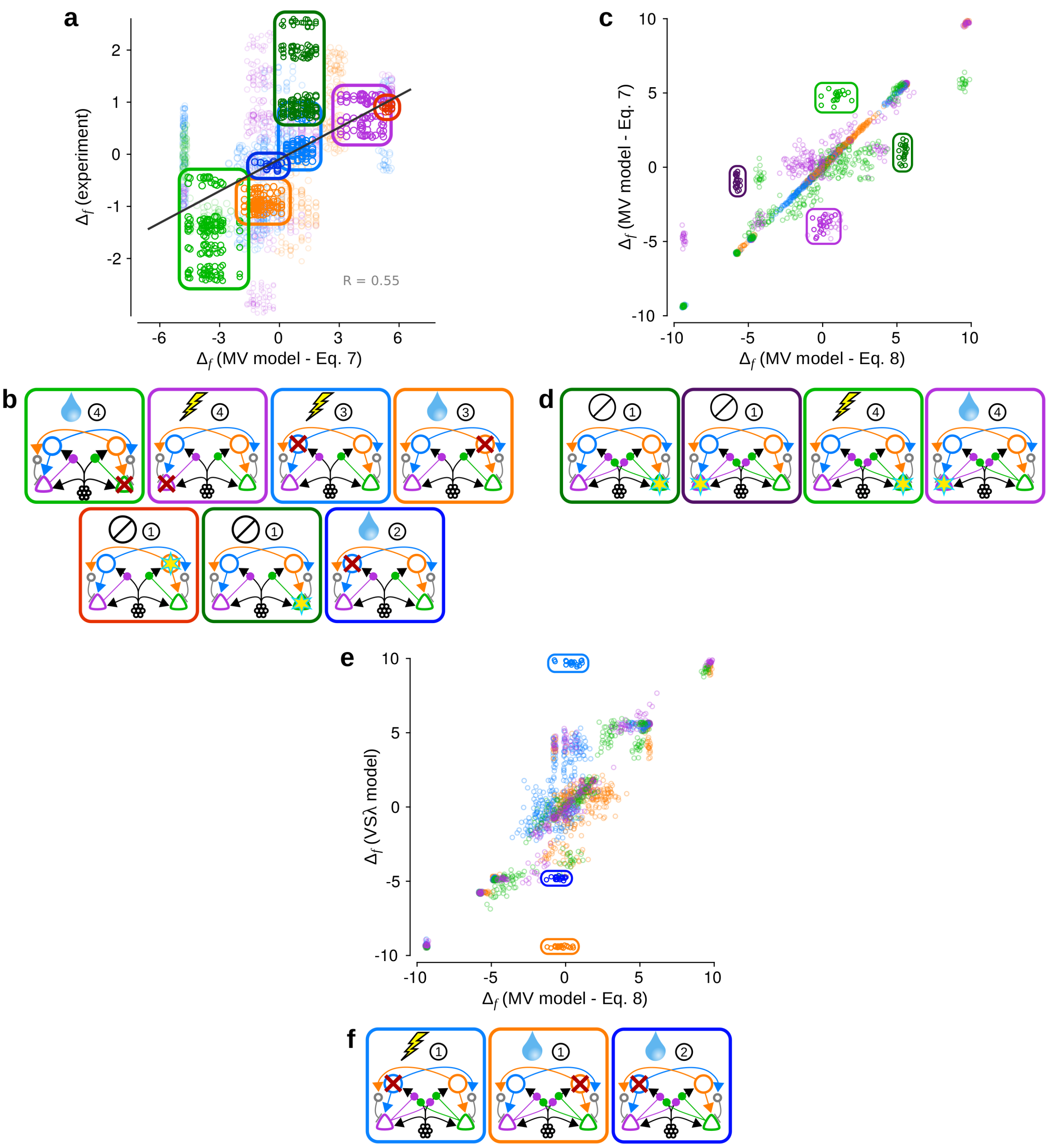

Supplementary Figure 7: Comparison of the different models for a broad range of conditioning experiments in *Drosophila*. Experimental data provided in Supplementary Table 2. **a** As with Fig. 5e in the main text, comparing Δ_f scores from experiments with those from the MV model, operating with the plasticity rule in Eq. 7. The dark green highlighted data corresponds to the dark green highlighted data in **c**. Solid grey line is a weighted least square linear fit with correlation coefficient $R = 0.55$ (0.51, 0.58) ($p < 10^{-4}$ using a permutation test; 95% confidence interval in parentheses using bootstrapping; $n = 92$). Each data point corresponds to a single Δ_f computed for a batch of 50 simulation runs, and for one pool of experiments using the same intervention from a single study. **b** Examples of the simulated interventions, as in Fig. 5c in the main text. **c** Comparison of Δ_f scores for the MV model operating with a plasticity rule according to either Eq. 8 (horizontal axis) or Eq. 7 (vertical axis). Both models correspond well for most experimental interventions, especially when interventions are applied to MBONs. Highlighted are four interventions (shown in **d** that help distinguish the two plasticity rules. **d** Description of the four interventions highlighted in **c**. Dark green: stimulating D_+ during the CS+ training only in the absence of other reinforcement. Dark purple: stimulating D_- during the CS+ training only in the absence of other reinforcement. Light green: stimulating D_+ throughout an aversive conditioning experiment. Light purple: stimulating D_- throughout an appetitive conditioning experiment. **e** Comparison of Δ_f scores for the VSλ model and the MV model using the plasticity rule in Eq. 8. Both models correspond well for most experimental interventions. Highlighted are three interventions (described in **f** that help distinguish the two models. Dark blue highlighted data corresponds to the dark blue highlighted data in **a** and in Fig. 5d-e in the main text. **f** Description of the three interventions highlighted in **e**. Light blue: blocking M_+ during CS+ training only in an aversive conditioning experiment. Orange: blocking M_- during CS+ training only in an appetitive conditioning experiment. Dark blue: blocking M_+ throughout training (CS+ and CS−) in an appetitive conditioning experiment. Source data are provided in the Supplementary Data 1 file. Abbrev.: MV (mixed valence); VSλ (valence specific with λ set point).

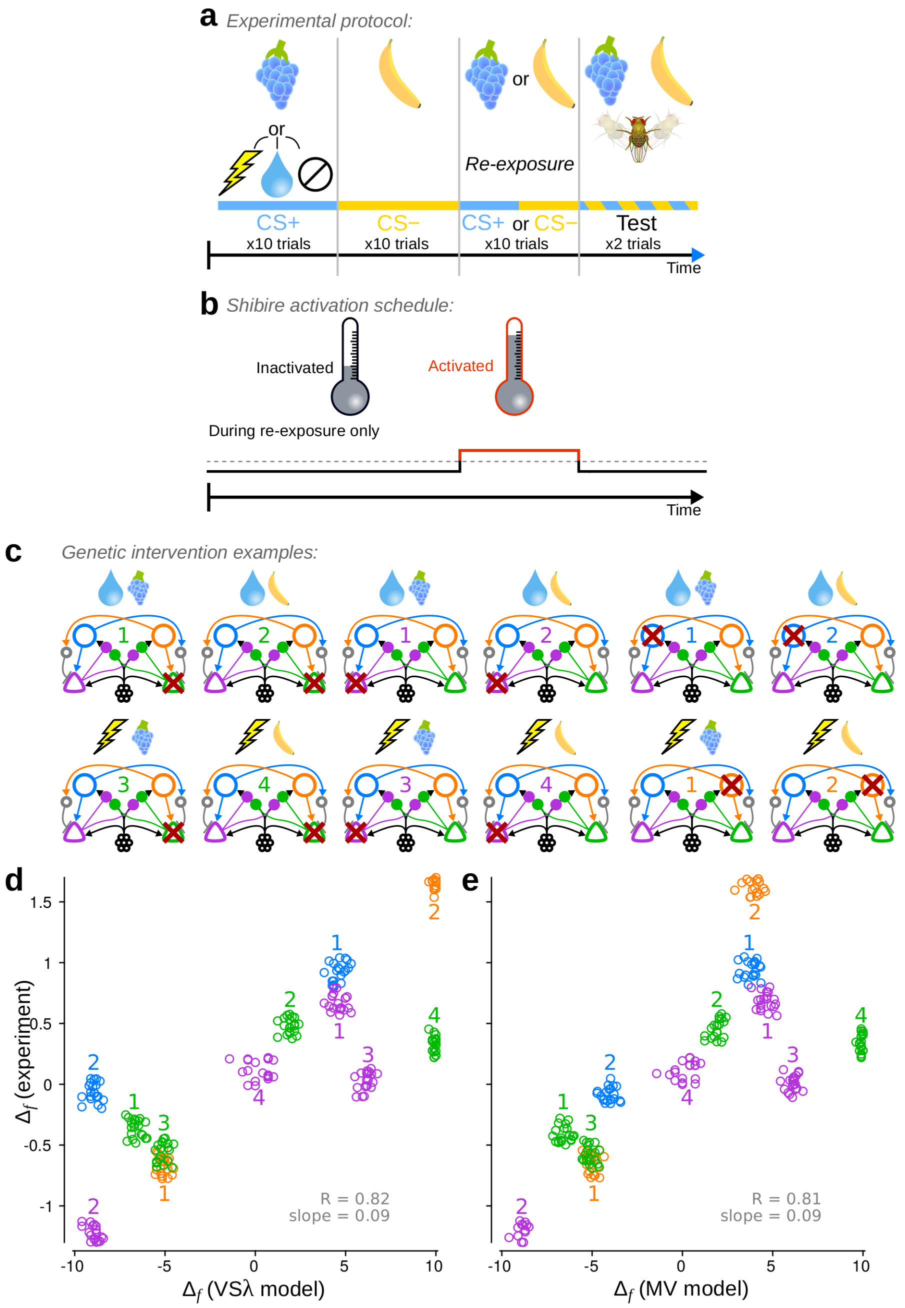

Supplementary Figure 8: Comparison of the VSλ and MV models with the re-evaluation experiments of Felsenberg et al. [11; 15]. Experimental data provided in Supplementary Table 3. **a** Schematic of the experimental paradigm. For the re-evaluation simulations, the same conditioning protocol as in Fig. 5 in the main text is used: a CS$+$ is paired with a US for 10 trials, and a CS$-$ is presented without a US for another 10 trials. However, before the final 2 test trials, either the CS$+$ or CS$-$ is re-exposed, without the US, for another 10 trials. In control simulations, re-exposure to the CS$+$ results in extinction of the memory: the reinforcement predictions (RPs) are re-learned such that $\hat{m}_{\mathrm{CS}+} \simeq 0$. **b** Interventions are also simulated in addition to the controls. For intervention simulations, either D$_+$, D$_-$, M$_+$ or M$_-$ is blocked during the re-exposure phase. **c** Examples of all the simulated interventions, which were also employed in the Felsenberg et al. experiments. Water drops denote an appetitive US ($r_+ = 1$, $r_- = 0$), thunderbolts denote an aversive US ($r_+ = 0$, $r_- = 1$). Grapes denote re-exposure to the CS$+$, bananas denote re-exposure to the CS$-$. Red crosses denote which neuron was blocked. Coloured numbers match each protocol with a cluster of data points in panels **d**-**e**. **d**-e Comparison of Δ_f scores between the Felsenberg et al. experiments and either the VSλ model (**d**) or the MV model (**e**; using the plasticity rule in Eq. 8). With the exception of small differences between two of the protocols, both models produce very similar results. Weighted least square linear fits yield correlation coefficients $R = 0.82$ (0.78, 0.89) for the VSλ model, and $R = 0.81$ (0.76, 0.87) for the MV model ($p < 10^{-4}$ for both models using a permutation test; 95% confidence intervals in parentheses using bootstrapping; $n = 12$). Each data point corresponds to a single Δ_f computed for a batch of 50 simulation runs, and for one pool of experiments using the same intervention from a single study. Source data are provided in the Supplementary Data 2 file. Abbrev.: CS$+/-$ (conditioned stimulus with/without reinforcement); MV (mixed valence); VSλ (valence specific with λ set point).

Supplementary Table

A	B	C	D	E	F	G	H	I	J
**DAN Valence**	**Effect of DAN on plasticity**	**Reward valence**	**Input from reward**	**MBON valence**	**MBON outputs**	**IS EFFECTIVE?**	**IS STABLE?**	**IS LTP?**	**CAN LEARN?**
+1: appetitive	+1: potentiation	+1: positive	+1: excitatory	+1: approach	+1: excitatory	B x C x D x E x F > 0	D + (B x D x F) = 0	B x D > 0	G AND H AND I
-1: aversive	-1: depression	-1: negative	-1: inhibitory	-1: avoidance	-1: inhibitory				
1	1	1	1	1	1	TRUE	FALSE	TRUE	FALSE
1	1	1	1	1	-1	FALSE	TRUE	TRUE	FALSE
1	1	1	1	-1	1	FALSE	FALSE	TRUE	FALSE
1	1	1	1	-1	-1	TRUE	TRUE	TRUE	TRUE
1	1	-1	-1	1	1	TRUE	FALSE	FALSE	FALSE
1	1	-1	-1	1	-1	FALSE	TRUE	FALSE	FALSE
1	1	-1	-1	-1	1	FALSE	FALSE	FALSE	FALSE
1	1	-1	-1	-1	-1	TRUE	TRUE	FALSE	FALSE
1	-1	1	1	1	1	FALSE	TRUE	FALSE	FALSE
1	-1	1	1	1	-1	TRUE	FALSE	FALSE	FALSE
1	-1	1	1	-1	1	TRUE	TRUE	FALSE	FALSE
1	-1	1	1	-1	-1	FALSE	FALSE	FALSE	FALSE
1	-1	-1	-1	1	1	FALSE	TRUE	TRUE	FALSE
1	-1	-1	-1	1	-1	TRUE	FALSE	TRUE	FALSE
1	-1	-1	-1	-1	1	TRUE	TRUE	TRUE	TRUE
1	-1	-1	-1	-1	-1	FALSE	FALSE	TRUE	FALSE
-1	1	1	-1	1	1	FALSE	FALSE	FALSE	FALSE
-1	1	1	-1	1	-1	TRUE	TRUE	FALSE	FALSE
-1	1	1	-1	-1	1	TRUE	FALSE	FALSE	FALSE
-1	1	1	-1	-1	-1	FALSE	TRUE	FALSE	FALSE
-1	1	-1	1	1	1	FALSE	FALSE	TRUE	FALSE
-1	1	-1	1	1	-1	TRUE	TRUE	TRUE	TRUE
-1	1	-1	1	-1	1	TRUE	FALSE	TRUE	FALSE
-1	1	-1	1	-1	-1	FALSE	TRUE	TRUE	FALSE
-1	-1	1	-1	1	1	TRUE	TRUE	TRUE	TRUE
-1	-1	1	-1	1	-1	FALSE	FALSE	TRUE	FALSE
-1	-1	1	-1	-1	1	FALSE	TRUE	TRUE	FALSE
-1	-1	1	-1	-1	-1	TRUE	FALSE	TRUE	FALSE
-1	-1	-1	1	1	1	TRUE	TRUE	FALSE	FALSE
-1	-1	-1	1	1	-1	FALSE	FALSE	FALSE	FALSE
-1	-1	-1	1	-1	1	FALSE	TRUE	FALSE	FALSE
-1	-1	-1	1	-1	-1	TRUE	FALSE	FALSE	FALSE

Supplementary Table 1: Criteria for unbounded, stable learning of reinforcement predictions. Here, we tabulate the properties of DANs, MBONs, and synaptic plasticity that modulate learning. Columns A-F describe the different properties, each of which takes a value of either $+1$ or -1, and each row provides a unique combination those properties. Note that column A is determined by the product of values in columns C and D. Columns G-I determine whether or not each of three criteria, all of which are required for learning, are satisfied by the particular combination of DAN, MBON, and plasticity properties. These criteria are: i) that learning induces the expected change in MBON firing rate and thus the expected change in behaviour; ii) that learning is stable, so that, for example, the addition of excitatory reinforcement information is offset after learning by the depression of feedback excitation or the potentiation of feedback inhibition. The condition under which each criteria is satisfied is determined by the expression in the second row, which states how the property values in columns B-F must be combined. Column J determines whether or not learning can occur. Only four combinations of properties (highlighted in blue) enable learning, and each one contributes to the MV model.

Supplementary References

[1] Schultz, W. Neuronal Reward and Decision Signals: From Theories to Data. *Physiological Reviews* **95**, 853–951 (2015).

[2] Riemensperger, T., Völler, T., Stock, P., Buchner, E. & Fiala, A. Punishment prediction by dopaminergic neurons in Drosophila. *Current Biology* **15**, 1953–1960 (2005).

[3] Sutton, R. S. Learning to Predict by the Methods of Temporal Differences. *Machine Learning* **3**, 9–44 (1988).

[4] Montague, P. R., Dayan, P. & Sejnowski, T. J. A framework for mesencephalic dopamine systems based on predictive Hebbian learning. *Journal of Neuroscience* **16**, 1936–1947 (1996).

[5] Schultz, W., Dayan, P. & Montague, P. R. A neural substrate of prediction and reward. *Science* **275**, 1593–1599 (1997).

[6] Rescorla, R. A. & Wagner, A. R. A theory of Pavlovian conditioning: variantions in the effectiveness of reinforcement and nonreinforcement. In Black, A. H. & Prokasy, W. F. (eds.) *Classical conditioning II: current research and theory*, 64–99 (Appleton-Century-Crofts, New York: Appleton-Century-Crofts, 1972).

[7] Dylla, K. V., Raiser, G., Galizia, C. G. & Szyszka, P. Trace Conditioning in Drosophila Induces Associative Plasticity in Mushroom Body Kenyon Cells and Dopaminergic Neurons. *Frontiers in Neural Circuits* **11**, 42 (2017).

[8] Mao, Z. & Davis, R. L. Eight different types of dopaminergic neurons innervate the Drosophila mushroom body neuropil : anatomical and physiological heterogeneity MATERIALS AND METHODS. *Front. Neural Circuits* **3**, 1–17 (2009).

[9] Burke, C. J. *et al.* Layered reward signalling through octopamine and dopamine in Drosophila. *Nature* **492**, 433–437 (2012).

[10] Liu, C. *et al.* A subset of dopamine neurons signals reward for odour memory in Drosophila. *Nature* **488**, 512–516 (2012).

[11] Felsenberg, J., Barnstedt, O., Cognigni, P., Lin, S. & Waddell, S. Re-evaluation of learned information in Drosophila. *Nature* **544**, 240–244 (2017).

[12] Eschbach, C. *et al.* Recurrent architecture for adaptive regulation of learning in the insect brain. *Nature Neuroscience* **23** (2020).

[13] Technau, G. & Heisenberg, M. Neural reorganization during metamorphosis of the corpora pedunculata in Drosophila melanogaster. *Nature* **295**, 405–407 (1982).

[14] Wang, Y. *et al.* Stereotyped Odor-Evoked Activity in the Mushroom Body of Drosophila Revealed by Green Fluorescent Protein-Based Ca2+ Imaging. *Journal of Neuroscience* **24**, 6507–6514 (2004).

[15] Felsenberg, J. *et al.* Integration of Parallel Opposing Memories Underlies Memory Extinction. *Cell* **175**, 709–722.e15 (2018).
