# Supplementary material for: Learning with reinforcement prediction errors in a model of the Drosophila mushroom body
Source: Nat Commun. 2021 May 7;12:2569. doi: 10.1038/s41467-021-22592-4 (PMC8105414; doi:10.1038/s41467-021-22592-4)
Supplement: Supplementary file 2 — Description of Additional Supplementary Files [file 41467_2021_22592_MOESM2_ESM.docx]

**Description of Additional Supplementary Files**

**Supplementary Data 1:** Experimental data compiled from previous studies, used in Fig. 5 and Supplementary Fig. S7.

**Supplementary Data 2:** Experimental data compiled from previous studies, used in Supplementary Fig. S8
