# Supplementary material for: Learning with reinforcement prediction errors in a model of the Drosophila mushroom body
Source: Nat Commun. 2021 May 7;12:2569. doi: 10.1038/s41467-021-22592-4 (PMC8105414; doi:10.1038/s41467-021-22592-4)
Supplement: Supplementary file 6 — Reporting Summary [file 41467_2021_22592_MOESM6_ESM.pdf]

nature research

Corresponding author(s): James Bennett

Last updated by author(s): Jan 5, 2021

Reporting Summary

Nature Research wishes to improve the reproducibility of the work that we publish. This form provides structure for consistency and transparency in reporting. For further information on Nature Research policies, see our Editorial Policies and the Editorial Policy Checklist.

Statistics

For all statistical analyses, confirm that the following items are present in the figure legend, table legend, main text, or Methods section.

n/a	Confirmed	
[ ]	[x]	The exact sample size (*n*) for each experimental group/condition, given as a discrete number and unit of measurement
[x]	[ ]	A statement on whether measurements were taken from distinct samples or whether the same sample was measured repeatedly
[x]	[ ]	The statistical test(s) used AND whether they are one- or two-sided<br>*Only common tests should be described solely by name; describe more complex techniques in the Methods section.*
[ ]	[x]	A description of all covariates tested
[ ]	[x]	A description of any assumptions or corrections, such as tests of normality and adjustment for multiple comparisons
[ ]	[x]	A full description of the statistical parameters including central tendency (e.g. means) or other basic estimates (e.g. regression coefficient) AND variation (e.g. standard deviation) or associated estimates of uncertainty (e.g. confidence intervals)
[x]	[ ]	For null hypothesis testing, the test statistic (e.g. *F*, *t*, *r*) with confidence intervals, effect sizes, degrees of freedom and *P* value noted<br>*Give P values as exact values whenever suitable.*
[x]	[ ]	For Bayesian analysis, information on the choice of priors and Markov chain Monte Carlo settings
[x]	[ ]	For hierarchical and complex designs, identification of the appropriate level for tests and full reporting of outcomes
[ ]	[x]	Estimates of effect sizes (e.g. Cohen's *d*, Pearson's *r*), indicating how they were calculated

Our web collection on statistics for biologists contains articles on many of the points above.

Software and code

Policy information about availability of computer code

Data collection	All simulation code was written in Matlab and has been tested up to version 2019a, It is freely available on the GitHub repository at https://github.com/BrainsOnBoard/paper_RPEs_in_drosophila_mb.
Data analysis	All code for analysing simulation results was written in Matlab and has been tested up to version 2019a. It is freely available on the GitHub repository at https://github.com/BrainsOnBoard/paper_RPEs_in_drosophila_mb.

For manuscripts utilizing custom algorithms or software that are central to the research but not yet described in published literature, software must be made available to editors and reviewers. We strongly encourage code deposition in a community repository (e.g. GitHub). See the Nature Research guidelines for submitting code & software for further information.

Data

Policy information about availability of data

All manuscripts must include a data availability statement. This statement should provide the following information, where applicable:

- Accession codes, unique identifiers, or web links for publicly available datasets
- A list of figures that have associated raw data
- A description of any restrictions on data availability

All experimental data was lifted from the data plotted in the cited publications. No additional experimental data was generated in this work. This data is collated and freely available at https://github.com/BrainsOnBoard/paper_RPEs_in_drosophila_mb.

Field-specific reporting

Please select the one below that is the best fit for your research. If you are not sure, read the appropriate sections before making your selection.

- [x] Life sciences
- [ ] Behavioural & social sciences
- [ ] Ecological, evolutionary & environmental sciences

For a reference copy of the document with all sections, see nature.com/documents/nr-reporting-summary-flat.pdf

Life sciences study design

All studies must disclose on these points even when the disclosure is negative.

Sample size	When comparing changes in performance index between simulations and previously published experiments, we equated a single run of the simulation with an individual animal. In experiments, each sample comprised behavioural data from approximately 50 individual flies, and approximately 20 samples were used. Thus, in our simulations, each performance index was calculated from 50 repeated runs of a given simulation, and this in turn was repeated 20 times, resulting in 1000 simulation runs per experimental protocol design. For simulations using multi-alternative forced choice tasks, we used 100 simulation runs per number of alternative choices. This was a heuristic choice that ensured means and standard deviations had converged.
Data exclusions	No data was excluded from our analyses. We used Matlab's built in robustfit function for computing the correlation coefficient between simulated and experimental results. This function weights each data point to help minimise the influence of outliers.
Replication	Each simulation run was deterministic. Repeated runs of a simulation were differentiated using different random noise sequences. When comparing changes in performance index between simulations and previously published experiments, each experimental protocol was simulated using 1000 runs, as described for the calculation of sample sizes above. For figures illustrating model behaviour e.g. in the firing rates of different neurons (for example Fig. 2), we repeated simulations 10 times. We deemed sufficient to show the consistency in the model behaviour.
Randomization	Random allocation was not required because of the computational nature of this work. Randomness was established as different sources of noise in the simulations.
Blinding	Blinding was not required because of the computational nature of this work.

Reporting for specific materials, systems and methods

We require information from authors about some types of materials, experimental systems and methods used in many studies. Here, indicate whether each material, system or method listed is relevant to your study. If you are not sure if a list item applies to your research, read the appropriate section before selecting a response.

Materials & experimental systems

n/a	Involved in the study
[x]	[ ] Antibodies
[x]	[ ] Eukaryotic cell lines
[x]	[ ] Palaeontology and archaeology
[x]	[ ] Animals and other organisms
[x]	[ ] Human research participants
[x]	[ ] Clinical data
[x]	[ ] Dual use research of concern

Methods

n/a	Involved in the study
[x]	[ ] ChIP-seq
[x]	[ ] Flow cytometry
[x]	[ ] MRI-based neuroimaging
